# Body fluids and salt metabolism - Part II

**DOI:** 10.1186/1824-7288-36-78

**Published:** 2010-12-13

**Authors:** Mattia Peruzzo, Gregorio P Milani, Luca Garzoni, Laura Longoni, Giacomo D Simonetti, Alberto Bettinelli, Emilio F Fossali, Mario G Bianchetti

**Affiliations:** 1 Department of Pediatrics, Bellinzona and Mendrisio, and University of Bern, Switzerland; 2 Emergency Unit, Clinica Pediatrica De Marchi, Foundation IRCCS Ca' Granda Ospedale Maggiore Policlinico, Milano, Italy; 3 Department of Pediatrics, San Leopoldo Mandic Hospital, Merate-Lecco, Italy; 4 Pediatric Nephrology, University of Bern, Switzerland

## Abstract

There is a high frequency of diarrhea and vomiting in childhood. As a consequence the focus of the present review is to recognize the different body fluid compartments, to clinically assess the degree of dehydration, to know how the equilibrium between extracellular fluid and intracellular fluid is maintained, to calculate the effective blood osmolality and discuss both parenteral fluid maintenance and replacement.

## Introduction

The first part of this review, published some months ago, outlined the physiology of the body fluid compartments, dehydration and extracellular fluid volume depletion [1]. The second part will focus the causes underlying dysnatremia and, more importantly, both the parenteral hydration and the management of dysnatremia.

## Dysnatremia

Under normal conditions, blood sodium concentrations are maintained within the narrow range of 135-145 mmol/L despite great variations in water and salt intake. Sodium and its accompanying anions, principally chloride and bicarbonate, account for 90% of the extracellular effective osmolality. The main determinant of the sodium concentration is the plasma water content, itself determined by water intake (thirst or habit), "insensible" losses, and urinary dilution. The last of these is under most circumstances crucial and predominantly determined by vasopressin. In response to this hormone, concentrated urine is produced by water reabsorption across the renal tubules. Dysnatremias produce signs and symptoms secondary to central nervous system dysfunction. While hyponatremia may induce brain swelling, hypernatremia may induce brain shrinkage, yet the clinical features elicited by opposite changes in tonicity are remarkably similar [2-7].

## Hyponatremia

### Introduction

**Figure 1:**
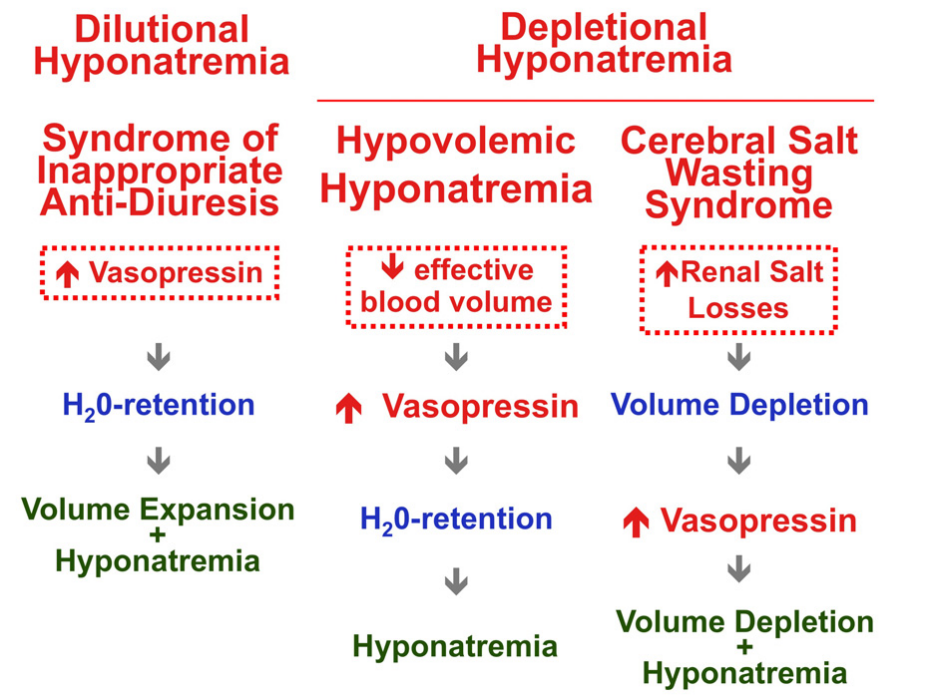
**Mechanisms underlying hypotonic hyponatremia**. In most cases (middle panel) hyotonic hyponatemia results from a low effective arterial blood volume and is termed hypovolemic (or depletional) hyponatremia. The term syndrome of appropriate anti-diuresis has also been used to denote this condition. In childhood diarrhea, vomiting and febrile infections are the most common cause of hypovolemic hyponatremia. Persistently high levels of vasopressin or, exceptionally, an increased renal response to vasopressin cause the syndrome of inappropriate anti-diuresis (left panel), which is less frequent than the syndrome of appropriate anti-diuresis (hypovolemic or depletional hyponatremia). A peculiar form of depletional hyponatremia sometimes develops in patients with cerebral disease that mimics all of the findings in the syndrome of inappropriate anti-diuresis, except that renal salt-wasting is the primary defect with the ensuing volume depletion leading to a secondary rise in release of antidiuretic hormone (right panel). The ultimate causes of the three different conditions are "bordered".

Hyoponatremia [4,6] is classified (Figure 1 left and middle panel) according to the extracellular fluid volume status, as either hypovolemic (= depletional) or normo- hypervolemic (= dilutional). Vasopressin is released both in children with low effective arterial blood volume, by far the most common cause of hyponatremia in everyday clinical practice, as well as in those with normo-hypervolemic hyponatremia [8]. In hypovolemic hyponatremia vasopressin release is triggered by the low effective arterial blood volume (this condition has been called by some syndrome of appropriate anti-diuresis). In dilutional hyponatremia [8] the primary defect is euvolemic, inappropriate increase in circulating vasopressin levels (this condition is also termed syndrome of inappropriate anti-diuresis).

Assessing the cause of hyponatremia may be straightforward if an obvious cause is present (for example in the setting of vomiting or diarrhea) or in the presence of a clinical evident extracellular fluid volume depletion. Sometimes, however, distinguishing hypovolemic from normo- hypervolemic hyponatremia may not be straightforward. In such cases, further laboratory investigations are warranted [4,6,8]:

a) the urine spot sodium and the fractional sodium clearance are helpful in patients in whom volume status is difficult to assess, as patients with dilutional hyponatremia have a urinary sodium > 30 mmol/L (and fractional sodium clearance > 0.5 × 10^-2^), whereas those with extracellular fluid volume depletion (unless the source is renal) will have a urinary sodium < 30 mmol/L (and fractional sodium clearance < 0.5 × 10^-2^). Since effective blood osmolality is mostly low in hyponatremia, and urine is less than maximally dilute (inappropriately concentrated), blood and urine osmolalities, although usually measured, are rarely discriminant.

b) in hypovolemic hyponatremia the urine spot sodium concentration and the fractional sodium clearance allow the distinction between extrarenal (sodium < 30 mmol/L; fractional sodium clearance < 0.5 × 10^-2^) and renal (sodium > 30 mmol/L; fractional sodium clearance > 0.5 × 10^-2^) salt loss.

A decrease in sodium concentration and effective blood osmolality causes movement of water into brain cells and results in cellular swelling and raised intracranial pressure. Nausea and malaise are typically seen when sodium level acutely falls below 125-130 mmol/L. Headache, lethargy, restlessness, and disorientation follow, as the sodium concentration falls below 115-120 mmol/L. With severe and rapidly evolving hyponatremia, seizure, coma, permanent brain damage, respiratory arrest, brain stem herniation, and death may occur. In more gradually evolving hyponatremia, the brain self regulates to prevent swelling over hours to days by transport of, firstly, sodium, chloride, and potassium and, later, solutes like glutamate, taurine, myoinositol, and glutamine from intracellular to extracellular compartments. This induces water loss and ameliorates brain swelling, and hence leads to few symptoms in subacute and chronic hyponatremia [4,6,8].

### Evaluating the cause

In normovolemic subjects, the primary defense against developing hyponatremia is the ability to dilute urine and excrete free-water. Rarely is excess ingestion of free-water alone the cause of hyponatremia. It is also rare to develop hyponatremia from excess urinary sodium losses in the absence of free-water ingestion. In order for hyponatremia to develop it typically requires a relative excess of free-water in conjunction with an underlying condition that impairs the ability to excrete free-water. Renal water handling is primarily under the control of vasopressin, which is released from the posterior pituitary and impairs water diuresis by increasing the permeability to water in the collecting tubule.

There are osmotic, hemodynamic and non-hemodynamic stimuli for release of vasopressin. In most cases, hyponatremia develops when the body attempts to preserve the extracellular fluid volume at the expense of circulating sodium (therefore, a hemodynamic stimulus for vasopressin production overrides an inhibitory effect of hyponatremia). However, there are further stimuli for production of vasopressin in hospitalized children that make virtually any hospitalized patient at risk for hyponatremia (Table 1).

**Table 1:** Causes of hypotonic hyponatremia in childhood.

Hypovolemic	Normovolemic (or hypervolemic)
**Intestinal salt loss**	**Increased body water**
- Diarrheal dehydration	- Parenteral hypotonic solutions
- Vomiting, gastric suction	- Exercise-associated hyponatermia
- Fistulae	- Habitual (and psychogenic) polydipsia
- Laxative abuse	
**Transcutaneous salt loss**	Non osmolar release of antidiuretic hormones*
- Cystic fibrosis	- Cardiac failure
- Endurance sport	- Sever liver disease (mostly cirrhosis)
	- Nephrotic syndrome
	- Glucocorticoid deficiency
	- Drugs causing renal water retention
	- Hyopthyroidism^Δ^
**Renal sodium loss**	**Syndrome of inappropriate anti-diuresis**
- Mineralocorticoid deficiency (or resistance)	- Classic syndrome of inappropriate secretion of antidiuretic hormone
- Diuretics	- Hereditary nephrogenic syndrome of inappropriate anti-dieresis
- Salt wasting renal failure	
- Salt wasting tubulopathies (including Bartter syndromes, Gitelman syndrome, and De Toni-Debré-Fanconi syndrome)	
- Cerebral salt wasting	
**Perioperative **(e.g.: preoperative fasting, vomiting, third space losses)	**Reduced renal water loss**
	- Chronic renal failure
	- Oliguric acute renal failure
**Third space losses **(e.g.: burns, major septic shock, surgery)	

* Effective arterial blood volume mostly reduced; ^Δ ^evidence supporting this association rather poor.

Some special causes of hypotonic hyponatremia deserve some further discussion.

**Figure 2:**
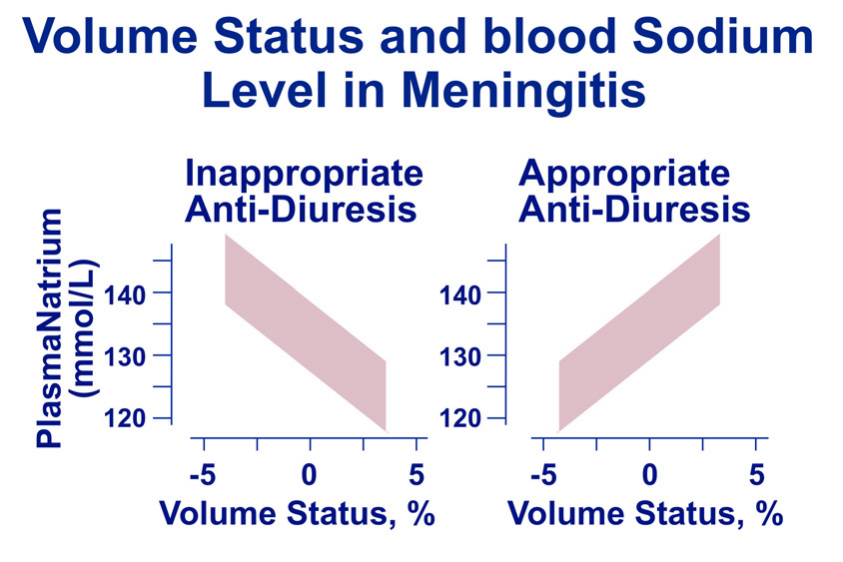
**Hypotetical diagrams depicting the relationship between initial state of hydration and plasma sodium in acute meningitis (and respiratory infectious diseases)**. It has been traditionally assumed that hyponatremia is due to inappropriate anti-diuresis (left panel). On the contrary, most recent data indicate that hyponatremia is due to appropriate, volume-dependent anti-diuresis (right panel).

**• Hospital-acquired hyponatremia **is most often seen in the postoperative period or in association with a reduced effective circulating volume [4,6]. More rarely hospital-acquired hyponatremia is seen in association with the syndrome of inappropriate anti-diuresis [8], which is caused either by elevated activity of vasopressin (80-90 percent of the cases) or by hyperfunction of its renal (= V2) receptor (10-20 pecent of the cases), independently of increased effective blood osmolality and hemodynamic stimulus (i.e.: reduced effective circulating volume). It is currently assumed that this condition results not only from dilution of the blood by free-water but also from inappropriate natriuresis [8]. The syndrome of inappropriate anti-diuresis (Figure 1 middle panel) should be suspected in any child with hyponatremic hypotonia, a urine osmolality above 100 mosmol/kg H_2_0, a normal fractional clearance of sodium (> 0.5 × 10^-2^), low normal or reduced uric acid level, low blood urea level and normal acid-base and potassium balance. The longstanding assumption that hypontremia [8-11] associated with meningitis and respiratory infectious diseases is caused by inappropriate anti-diuresis has not been substantiated by reports that adequately assessed the volume status (Figure 2).

Postoperative hyponatremia is a serious problem in children, which sometimes is caused by a combination of nonosmotic stimuli for release of antidiuretic hormone, such as pain, nausea, stress, narcotics, and edema-forming conditions [4,6,8]. However, subclinical depletion of the effective arterial blood volume and administration of hypotonic fluids are the most important causes of postoperative hyponatremia.

**• Desmopressin**, a synthetic analogue of the natural antidiuretic hormone, is used in central diabetes insipidus, in some bleeding disorders, in diagnostic urine concentration testing and especially in primary nocturnal enuresis with nocturnal polyuria. Desmopressin is generally regarded as a safe drug and adverse effects due to treatment are uncommon. Nonetheless, hyponatremic water intoxication leading to covulsions has been reported as a rare but potentially life threatening side effect of desmopressin therapy in enuretic children with high fluid intake during the day [12].

**• **Male infants have been recently described with hyponatremia and laboratory features consistent with release of vasopressin but who had no measurable circulating levels of this hormone. Genetic testing revealed gain-of-function mutations of the X-linked receptor gene that mediates the renal response to vasopressin, resulting in persistent activation of the receptor [8,13]. This very rare disease has been termed **hereditary nephrogenic syndrome of inappropriate anti-diuresis**: it represents a kind of mirror image of the X-linked nephrogenic diabetes insipidus, which results from loss-of-function genetic defects in the aforementioned renal receptor [8,13].

**• Cerebral salt wasting syndrome **is a peculiar form of depletional hyponatremia that sometimes occurs in patients with cerebral disease (Figure 1 right panel). It mimics the findings in the syndrome of inappropriate anti-diuresis, except that salt-wasting is the primary defect with the ensuing volume depletion leading to a secondary release of vasopressin [8,14]. It has been suggested that renal salt wasting of central origin results from increased secretion of a natriuretic peptide with subsequent suppression of aldosterone synthesis. The clinical distinction between cerebral salt wasting and inappropriate activity of vasopressin is not always simple to make since the true volume status is sometimes difficult to ascertain [8,14].

**• Endurance athletes **sometimes replace their dilute but sodium-containing sweat losses with excessive amounts of severely hypotonic solutions: the net effect is a reduction in the circulating sodium level (the effect is likely compounded by a reduced renal blood flow and glomerular filtration rate during exercise). Such individuals may also be taking non-steroidal anti-inflammatory drugs, which can impair the excretion of free water [4,6].

**• **A tendency towards low normal plasma sodium level is sometimes seen in children who drink excessively and present with polyuria and polydipsia [15]. Usually the problem is simply one of habit, particularly in infants who are attached to a bottle (= **habitual polydipsia**). Rarely, in childhood, polydispia is a symptom of significant psychopathology (= **psychogenic polydipsia**).

**• Diuretics**, mostly thiazides, and **drugs that block the renin-angiotensin-aldosterone system**, either converting enzyme inhibitors or sartans, make up a common cause of hyponatremia (Additional file 1: Table S1). More rarely, other drugs sometimes cause renal retention of fluids and therefore dilutional hyponatremia [16].

## Hypernatremia

### Introduction

Hypernatremia reflects a net water loss or a hypertonic sodium gain, with inevitable hypertonicity [3,5]. Severe symptoms are usually evident only with acute and large increases in sodium concentrations to above 160 mmol/L. Importantly, the sensation of thirst protecting against the tendency towards hypernatemia is absent or reduced in patients with altered mental status or with hypothalamic lesions and in infancy.

The cause of hypernatremia is almost always evident from the history. Determination of urine osmolality in relation to the effective blood osmolality and the urine sodium concentration helps if the cause is unclear. Patients with diabetes insipidus present with polyuria and polydipsia (and not hypernatremia unless thirst sensation is impaired). Central diabetes insipidus and nephrogenic diabetes insipidus may be differentiated by the response to water deprivation (failure to concentrate urine) followed by desmopressin, causing concentration of urine in patients with central diabetes insipidus.

Non-specific symptoms such as anorexia, muscle weakness, restlessness, nausea, and vomiting tend to occur early. More serious signs follow, with altered mental status, lethargy, irritability, stupor, or coma. Acute brain shrinkage can induce vascular rupture, with cerebral bleeding and subarachnoid hemorrhage [3].

### Evaluating the cause

Two mechanisms protect against developing hypernatremia (sodium 145 mmol/L or more) or increased effective blood osmolality: the ability to release vasopressin (and therefore to concentrate urine) and a powerful thirst mechanism. Release of vasopressin occurs when the effective blood osmolality exceeds 275-280 mosmol/kg H_2_O and results in maximally concentrated urine when the effective blood osmolality exceeds 290-295 mosmol/kg H_2_O [3,5]. Thirst, the second line of defense, provides a further protection against hypernatremia and increased effective osmolality. If the thirst mechanism is intact and there is unrestricted access to free-water, it is rare to develop sustained hypernatremia from either excess sodium ingestion or a renal concentrating defect (Table 2). Hypernatremia is primarily a hospital-acquired condition occurring in children who have restricted access to fluids. Most children with hypernatremia are debilitated by an acute or chronic disease, have neurological impairment, are critically ill or are born premature. Hypernatremia in the intensive care setting is common as these children are typically either intubated or moribund, and often are fluid restricted, receive large amounts of sodium as blood products or have renal concentrating defects from diuretics or renal dysfunction. The majority of hypernatremia results from the failure to administer sufficient free-water to children who are unable to care for themselves and have restricted access to fluids [2,3,5].

**Table 2:** Causes of hypernatremia in childhood.

Hypovolemic	Normovolemic	Hypervolemic
**Inadequate Intake**		
- Breast feeding hypernatremia	**Hypodypsia **(essential hypernatremia)	**Inappropriate intravenous fluids **(e.g.: hypertonic saline, NaHCO_3_)
- Poor access to water	**Hyperventilation**	**Salt poisoning **(accidental, deliberate)
- Altered thirst perception (uncosciousness, mental impairment)	**Fever**	**Primary aldosteronism **(and other conditions that cause low-renin hypertension)
		
**Intestinal salt loss **(diarrheal dehydration)		
		
**Renal water and salt loss**		
- Postobstructive polyuria		
- Diuretics		
- Diabetes insipidus		
- Medullary renal damage		

Two special causes of hypernatremia deserve some further discussion.

**• **A frequent cause of hypernatremia in the outpatient setting is currently **breastfeeding-associated hypernatremia**, which should more properly be labeled "not-enough-breastfeeding-associated hypernatremia" [17]. This condition occurs between days 7 and 15 in otherwise healthy term or near-term newborns of first-time mothers who are exclusively breast-fed. In all cases feeding had been difficult to establish and the volume of milk ingested was likely to have been low. The underlying problem is water deficiency: sodium concentration raises predominantly as a result of low volume intake and a loss of water, demonstrating that inadequate feeding is the cause of hypernatremic dehydration. Monitoring postnatal weight loss provides an objective assessment of the adequacy of nutritional intake allowing targeted support to those infants who fail to thrive or demonstrate excessive weight loss (10 percent or more of birth weight).

**• Diarrhea or vomiting **are a further reason of hypernatremia in the outpatient setting, but are much less common than in the past, presumably due to the advent of low solute infant formulas and the increased use and availability of oral rehydration solutions [2,3,5,18].

## Management

The discussion will exclusively focus some points of parenteral hydration and the management of hyponatremia with either V2 antidiuretic hormone receptor antagonists or urea.

### Parenteral hydration

#### • Maintenance and perioperative fluids

Intravenous maintenance fluids are designed to provide water and electrolyte requirements in a fasting patient. The prescription for intravenous maintenance fluids was originally described by Holliday more than 50 years ago [19], who rationalized a daily H_2_0 requirement of 1700-1800 ml/m^2 ^body surface area and the addition of 3 and 2 mmol/kg body weight of Na^+ ^and K^+ ^respectively (as it approximates the electrolyte requirements and urinary excretion in healthy infants). This is the basis for the traditional recommendation that hypotonic intravenous maintenance solutions are ideal for children [19]. In clinical practice the daily parenteral water requirement is calculated as given in Table 3 (left panel). This approach has been recently questioned considering the potential of these hypotonic solutions in determining hyponatremia and subsequently severe neurological sequelae [20-22]. Surgical patients appear the subgroup of pediatric patients with the highest risk to develop severe hyponatremia with the use of hypotonic intravenous solutions, likely because they tend to be hypovolemic. Furthermore, traditional maintenance fluid recommendations might be largely greater than actual water needs in children at risk of hyponatremia.

**Table 3:** Intravenous maintenance fluids designed to provide water and electrolyte requirements in a fasting patient.

	Holliday's recommendation	Current suggestion
Solution	5 percent dextrose in water supplemented with NaCl 3 mmol/kg body weight daily	Isotonic saline in 5 percent dextrose in water
Amount (ml/m^2 ^body surface area* daily)	1700-1800	1400-1500
Clinical practice	100 mL/kg body weight for a child weighing less than 10 kg^◇ ^+ 50 mL/kg for each additional kg up to 20 kg + 20-[25] mL/kg for each kg in excess of 20 kg	80 mL/kg body weight for a child weighing less than 10 kg^◇ ^+ 40 mL/kg for each additional kg up to 20 kg + 15-[20] mL/kg for each kg in excess of 20 kg

* the Mosteller's formula may be used to calculated the body surface area (in m^2^): $\sqrt{\frac{\text{height (cm)}\times \text{body wight (kg)}}{\text{3600}}}$; **◇ **in children weighing ≤5.0 kg the daily parenteral water requirement is 120 mL/kg body weight.

Both the recommendation originally described by Holliday and the most recent recommendation are given. The addition of KCl 2 mmol/kg body weight is also recommended.

More recente data [20-22] suggest that the prevention of hyponatremia should be obtained both by using isotonic (usually normal saline, which contains NaCl 9 g/L) or near-isotonic (usually lactate Ringer) solutions (Table 3 right panel) and by reducing the volume of maintenance fluid (approximately by 20 percent). Considering the potential of hypoglycemia in infancy, isotonic saline in 5 percent glucose in water (which contains approximately glucose 50 g/L and NaCl 9 g/L) seems to be the safest fluid composition in most children [20-22]. On the other side, we refrain from the uncritical, generalized adoption of this new standard care until rigorous trials confirming this suggestion have been made.

#### • Dehydration

Oral rehydration therapy is currently the treatment of choice for children with minimal, mild or moderate dehydration due to diarrheal diseases. However, in the practice of pediatric emergency medicine, intravenous rehydration is a commonly used intervention for these children [21,23].

Treatment approaches to parenteral rehydration in the hospitalized child vary. There are numerous ways to estimate the degree of dehydration (the "4-item 8-point rating scale" is currently widely recommended [1]) and especially to calculate fluid and electrolyte deficits, and to deliver the deficits to the patient. For many years, the traditional teaching was that 100 percent (or even less) replacement of the volume deficit should be accomplished during the first 24 hours of treatment. In recent years, the aim of treatment has generally been to accomplish a more rapid full repletion within 6 hours or less [21,23]. In many children with mild to moderate dehydration, especially those resistant to initial oral rehydration therapy, and in children with severe dehydration, we currently administer intravenous isotonic (or near isotonic) crystalloid solutions such as normal saline or lactate Ringer as repeated boluses of [10]-20 mL/kg body weight (administered over 20 to 60 minutes).

In children with diarrhea and vomiting reduced carbohydrate intake leads to free fatty acid breakdown, excess ketones, and an increased likelihood for continued nausea and vomiting. Consequently, some authorities have suggested (but so far not proven) that the use of a glucose containing isotonic solution (mostly the aforementioned isotonic saline in 5 percent glucose in water), which will stimulate insulin release, reduce free fatty acid breakdown, and therefore reduce treatment failure due to persisting nausea and vomiting [24].

The child with circulatory shock presents with a) increased heart rate and weak peripheral pulses, b) cold, pale and diaphoretic skin, and c) delayed capillary refill. The initial management recommended by the American Academy of Pediatrics includes the administration of a high concentration of oxygen (ensuring that 100 percent of the available arterial hemoglobin is oxygenated) and the fluid resuscitation with a 20 mL/kg body weight bolus of an isotonic crystalloid over 5-20 minutes (if the child fails to improve, at least 2 further boluses for a total of 60 mL/kg body weight are rapidly given). The most common error in the child with circulatory shock secondary to a diarrheal disease is the delayed or inadequate (i.e. with hypotonic crystalloid solution) fluid resuscitation.

**Figure 3:**
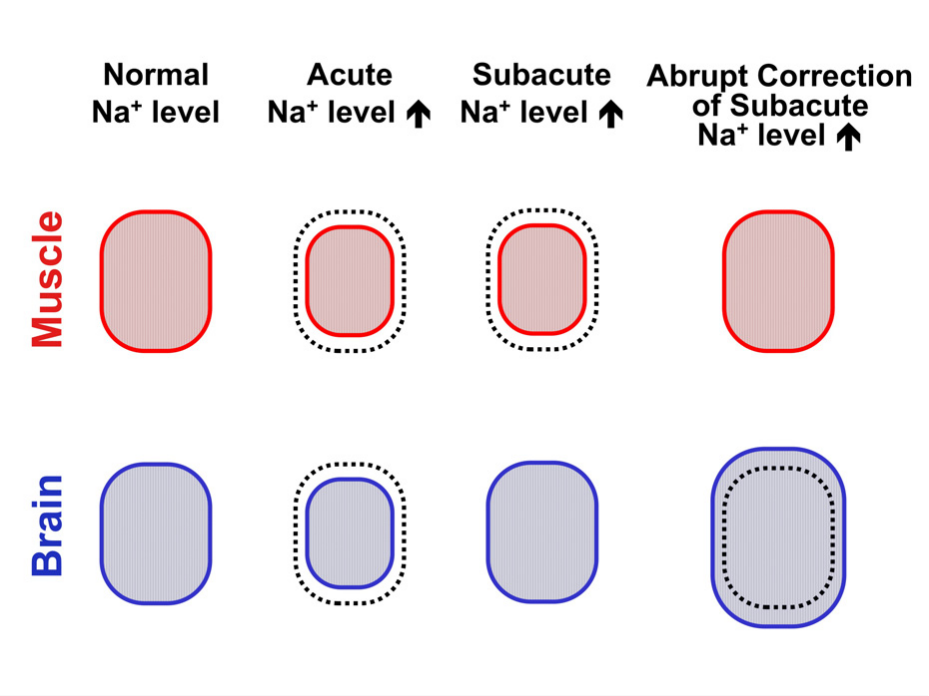
**Cell volume in acute or subacute hypernatremia and following rapid correction of hypernatremia**. When hypernatemia develops acutely, all cells are reduced in size (the degree of cell volume reduction reflects the degree of hypernatremia). When hypernatremia is present for 36-48 hours or more (= subacute hypernatremia), cell volume reduction persists in most cells, including muscle cells (upper panel). However, brain cells (and red blood cells) tend to restore their normal cell volume (lower panel). An abrupt normalization of sodium level in children with subacute or chronic hypernatremia pathologically increases the volume of brain cells (and red blood cells). Swelling of cells, which does not have serious consequences when it occurs in most organs, may have devastating consequences when it occurs in the brain (lower panel, right).

Children with hypernatremic dehydration are also hydrated parenterally with isotonic crystalloid solutions until diagnosis of the dyselectrolytemia, followed by slightly hypotonic solutions (e.g.: half-saline) in order to slowly correct circulating sodium level (abruptly correcting hypernatremia using a sodium free glucose solution creates an increased risk for the development of brain edema; Figure 3). In acute dysnatremic dehydration, sodium should be corrected slowly at a rate not exceeding 0.5 mmol/L per hour and no more than by 12 mmol/L per day. Subacute or chronic hypernatremia should be corrected even more slowly [3].

#### • Hydration in infectious diseases associated with a tendency towards hyponatremia

Fluid restriction has been widely advocated in the initial management of infectious diseases such as meningitis, pneumonia or bronchiolitis, which are often associated with a low sodium level. However, there is no evidence that fluid restriction is useful. Furthermore, hyponatremia results from appropriate, volume-dependent anti-diuresis in these disease conditions. In clinical practice, initial restoration of the intravascular space with an isotonic crystalloid followed by isotonic maintenance fluids 1400-1500 mL/m^2 ^body surface area daily (Table 3 right panel) are currently advised [8]. In cases presenting with overt hyponatremia frequent monitoring of electrolytes is also required with adjustments made as warranted by laboratory findings.

#### • Chronic hyponatremia

Chronic normovolemic (or hypervolemic) hyponatremia has been traditionally managed either by restricting water intake or by giving salt. An alternative may be the use of nonpeptide vasopressin receptor antagonists [25]. There are multiple receptors for vasopressin: the V1_a _receptors that mediate vasoconstriction, the V1_b _receptors that mediate adrenocorticotropin release, and the V2 receptors that mediate the antidiuretic response. Vaptans, oral V2 receptor antagonists, have been recently approved for the management of normovolemic and hypervolemic hyponatremia: these agents produce a selective water diuresis (without affecting sodium and potassium excretion) that raises the circulating sodium level [25]. No information is currently available with these agents in childhood.

Vaptans do not correct hyponatremia in patients affected with nephrogenic syndrome of inappropriate childhood anti-diuresis [8,13]. In these patients, a way to enhance water excretion is the oral administration of urea (dosage in adulthood: 30 g per day). This regimen, which may be effective because it causes simultaneously water diuresis and renal sodium retention, is well tolerated, and has been used chronically in ambulatory pediatric patients [8,26].

## Conclusions

In conclusion pediatricians must be aware of the changing epidemiology of dysnatremia in children with diarrhea (and vomiting) and in those hydrated parenterally with the hypotonic solutions recommended by Holliday. We recommend that clinicians consider more frequently the use of isotonic or near-isotonic crystalloid solutions both for replacement, i.e. to expand the extracellular fluid compartment, as well as for maintenance. Finally, recent data indicate that in meningitis and respiratory infections hyponatremia results from appropriate, volume-dependent anti-diuresis.

## Competing interests

The authors declare that they have no competing interests.

## Authors' contributions

MGB, AB and GDS wrote the first version of the manuscript. MP, GPM, LL and LG consistently revised the manuscript and prepared both the figures as well as the references. EFF revised the final version of the manuscript. All authors have read and approved the paper, have met the criteria for authorship as established by the International Committee of Medical Journals Editors, believe that the paper represents honest work, and are able to verify the validity of the content.

## Supplementary Material

Additional file 1 **Table S1**. Click here for file
