# Supplementary material for: Body fluids and salt metabolism - Part II
Source: Ital J Pediatr. 2010 Dec 13;36:78. doi: 10.1186/1824-7288-36-78 (PMC3022615; doi:10.1186/1824-7288-36-78)
Supplement: Additional file 1 — Table S1. [file 1824-7288-36-78-S1.DOC]

**Table S1. Drug-induced hyponatremia.**

**⬤ Diuretics** (thiazides more frequently than loop diuretics)

**⬤ Drugs blocking the renin-angiotensin-aldosterone system** (converting enzyme inhibitors or sartans)

**⬤ Antidiuretic drugs**

**⬤** 🡹 **water permeability of the renal collecting tubule:** arginine-vasopressin, vasopressin analogues like desmopressin, oxytocin

**⬤** 🡹 **antidiuretic hormone release,** 🡹 **antidiuretic hormone action:** carbamazepine, barbiturates, chlorpropamide, clofibrate, colchicine, nicotine, vincristine, cyclophosphamide

**⬤** 🡻 **synthesis of prostaglandins:** nonsteroidal anti-inflammatory drugs including salicylates, paracetamol

**⬤ Mechanism unknown:** haloperidol, amitryptiline, selective serotonin-reuptake inhibitors like fluoxetine, (narcotics like morphine*)

* evidence supporting the association between narcotics and antidiuresis is rather poor.
